# Happy Enough to Relax? How Positive and Negative Emotions Activate Different Muscular Regions in the Back - an Explorative Study

**DOI:** 10.3389/fpsyg.2021.511746

**Published:** 2021-05-31

**Authors:** Clara Scheer, Simone Kubowitsch, Sebastian Dendorfer, Petra Jansen

**Affiliations:** ^1^Institute of Sport Science, Faculty of Humanities, University of Regensburg, Regensburg, Germany; ^2^Laboratory for Biomechanics, Ostbayrische Technische Hoschschule Regensburg, Regensburg, Germany

**Keywords:** electromyography, muscle activity, emotion, sadness, happiness, embodiment

## Abstract

Embodiment theories have proposed a reciprocal relationship between emotional state and bodily reactions. Besides large body postures, recent studies have found emotions to affect rather subtle bodily expressions, such as slumped or upright sitting posture. This study investigated back muscle activity as an indication of an effect of positive and negative emotions on the sitting position. The electromyography (EMG) activity of six back muscles was recorded in 31 healthy subjects during exposure to positive and negative affective pictures. A resting period was used as a control condition. Increased muscle activity patterns in the back were found during the exposure to negative emotional stimuli, which was mainly measured in the lumbar and thorax regions. The positive emotion condition caused no elevated activity. The findings show that negative emotions lead to increased differential muscle activity in the back and thus corroborate those of previous research that emotion affects subtle bodily expressions.

## Introduction

Emotional reactivity and bodily expression are fundamental to react appropriately to the environment (Darwin, 1972). Emotional reactions are an important tool for social communication (Levenson, 2003). Until now, most studies have examined emotional expressions through muscle activation in the face. It is the Facial Action Coding-System (FACS) that extensively describes activated muscles for emotional expressions (Ekman and Friesen, 1978). However, more recent studies have examined the importance of bodily expressions of emotions and found them equally valid to facial expressions for social interaction (De Gelder, 2009). Some researchers have even proposed that bodily expressions are crucial for successful emotion recognition (Tracy and Robins, 2004). De Gelder and Van den Stock (2011) provided a set of whole body expressions (faces blurred) of four emotions (anger, fear, happiness, and sadness) and showed that sadness was most likely recognized, followed by fear. Above that, perceiving the whole body was found to be crucial for emotion recognition. Removed hands, for example, significantly reduced the recognition of anger and fear; whereas removed arms showed no decreased accuracy in emotion recognition (Ross and Flack, 2020).

Obviously, large body movements and facial expressions can be recognized by others. However, emotions have also been found to elicit more subtle physiological reactions. Some of the most well-known autonomic functions might be the change in heart rate, breathing, or skin color, or the appearance of sweat or goosebumps (Kret et al., 2020). Besides that, pupil dilation has been shown to respond to emotional expressions, such as angry faces, bodies, and aggressive contexts (Kret et al., 2013), and muscle activity, as proposed by Lelard et al. (2013). During the perception of painful pictures, the activity of the tibialis anterior muscle, situated lateral to the tibia, increased significantly. The authors conclude that the imagination of a painful situation increases muscle stiffness by leaning backward, which is suggested to be an expression of avoidance behavior. Another study that exposed participants to pictures of fearful and angry body expressions revealed that the muscle patterns needed for this specific emotional posture (upper trapezius descendens, anterior deltoid, biceps brachii, and triceps brachii bilaterally) would be activated in response to only observing it (Huis In‘t Veld et al., 2014a,b). Also, wrist and finger extensor muscles were shown to increase force production while the participants observed unpleasant images (Coombes et al., 2006). While subtle emotional expressions might not be consciously recognized by others, they tend to be mimicked by the interacting partner (i.e., pupil dilation) (Kret et al., 2014).

Embodiment theories have proposed a reciprocal relationship between emotional state and bodily reactions (Michalak et al., 2009). Depression patients, or with sadness induced participants, walk with decreased speed, arm swing, and vertical head movements, compared to healthy or happy participants (Michalak et al., 2009). Also, the sitting posture has been affected by emotions. Positive feelings cause a more upright sitting position, whereas emotionally negative feelings lead to a less upright sitting position (Oosterwijk et al., 2009). Previous research has found the sitting position and its adjustments to affect muscle activity in the trapezius, thoracic (Caneiro et al., 2010), and lumbar regions (Castanharo et al., 2014).

The influence of emotions on the body is based on the assumption that emotions modulate the readiness for actions of a person (Van Loon et al., 2010). According to this, positive emotions foster approach behavior, whereas negative emotions prime avoidance (Ackerley et al., 2017). There is a considerable amount of research that has indicated the effect of emotional stimuli on the behavioral response (for review, see Phaf et al., 2014). It decreases when consolidating a sad memory, whereas it increases when remembering a joyful or angry incident (Kang and Gross, 2015). Besides, the effect of emotions on the body is also known in the field of clinical research. For instance, negative emotions have been found to increase muscle tension in the back, which potentially aggravates chronic pain (Burns, 2006). Therefore, for this study, we chose to investigate back muscle activity as an indication of the effect of emotions on the sitting position. As other subtle emotional expressions have been found to be mimicked by others, we argue that there is also a potential for the sitting posture to be recognized (un)consciously.

The ability to recognize the emotional expressions of the face, or the body, requires sensorimotor simulation activity (Ross and Atkinson, 2020). This process has been proposed to provoke similar motor movements (Hawk et al., 2012) and enhances own emotional experience. The effect of experiencing to feel the emotional state of someone through observation is generally known as emotion contagion (Hatfield et al., 1993) and is proposed to occur with or without behavior mimicry, a spontaneous imitation of perceived motor movement (Ross and Atkinson, 2020). For the above-mentioned finding, sensorimotor simulation is a reasonable explanation. If the concept is true, however, body parts other than the observed ones should also respond. To address this question, Moody et al. (2018) presented angry and fearful faces while investigating the muscular activity of the face and forearm of participants. The results showed that muscle patterns are activated in the face; more importantly, however, also in the forearm, respective to the observed emotion. These results provide a promising avenue to understand the processing of perceived emotional expressions by simulation.

Pursuing these results, this study aims to deepen the understanding of the sensorimotor simulation process by investigating the effect of emotions on back muscle activity. First, we investigated the responsiveness of back muscles to positive and negative emotions, which were induced through emotional pictures and music. Second, we explored whether these emotions affect different muscular regions of the back (shoulder, thorax, and lumbar regions), which have been found to react to sitting positions (Caneiro et al., 2010; Castanharo et al., 2014) during inactivity. Above that, trunk movements have been suggested to be a good predictor to distinguish emotional expressions (de Meijer, 1989). The quality of the positive emotion condition approximates the emotion of *happiness*, and the quality of the negative emotion condition approximates the emotion of *sadness*. Since we were interested in the emotional effect caused by valence rather than the state of arousal, we selected *happiness* and *sadness* because they strongly differ along the dimension of valence but are equal according to their effect on arousal (Russell, 1980). Previous studies have found sadness and happiness to effectively affect body movements (Michalak et al., 2009; Kang and Gross, 2015). The combination of visual and musical stimuli was chosen to intensify the emotional impression.

According to the sensorimotor simulation hypothesis, we expect both positive and negative emotions to influence back muscle activity. Since emotional stressors have been found to affect the area of the trapezius and lumbar (Burns, 2006), and happiness and sadness have cognitively been associated with the areas of the upper and lower body (Nummenmaa et al., 2014), we expect increased muscle activity in the shoulder and lumbar region during positive and negative emotion induction.

## Materials and Methods

### Participants

Thirty-one healthy young adults (18 females and 13 males) volunteered and gave written informed consent to participate in the study (mean ± SD: age of females 20.8 ± 2.69; weight 61.6 ± 8.5 kg, height 168 ± 6 cm, body mass index (BMI) 21.9 ± 2.7 kg/m^2^; age of males 22.2 ± 1.92; weight 77 ± 6.8 kg, height 182 ± 5 cm, BMI 23.2 ± 1.5 kg/m^2^), and 90.3% was right-handed. They were instructed to refrain from caffeine for 4 h and from alcohol for 24 h before the experiment started, because of the influence of caffeine (Barry et al., 2005) and alcohol (Carpenter, 1875) on skin conductance. A questionnaire surveyed pharmaceutical drug intact, muscular, and psychological disorders. A participant was excluded because of recent lumbar disc herniation. The experimental procedure was approved by the ethical committee of the University of Regensburg and conducted according to the ethical guidelines of Helsinki.

### Measures

#### Skin Conductance

Skin conductance has been proven to increase with the state of arousal by several studies (e.g., Horslen and Carpenter, 2011). Skin conductance of the non-dominant hand was recorded using two 8-mm skin conductance flex/pro sensors (Ag/AgCl, SA9309M) attached to the medial phalanx of the third and fourth fingers. Prior to the attachment of the electrodes, the skin was prepared with a NaCl electrolyte cream as recommended (Fowles et al., 1981). Skin conductance was encoded in microsiemens by an eight-channel encoder (ProComp Infiniti; Thought Technology, Montreal, Quebec, Canada) with a frequency of 256 Hz and a band pass of 0.05–1 kHz.

#### Electromyography

EMG data were collected from 12 sites of the back: both sides of the M. trapezius pars descendens, the M. trapezius pars transversalis, the M. trapezius pars ascendens, which extend laterally at the upper spine from the occipital bone to the thoracic vertebra. The upper part supports the weight of the arm, and the middle and lower parts retract, rotate, and depress the scapula. The M. longissimus thoracis, between the sacrum and thoracic spines ensures elongation and leaning sideward of the thoracic and lumbar spine. The M. iliocostalis lumborum, between the ilium, sacrum and the 6th and 7th rib allows erection and stabilization of the spine. The M. multifidus sacrales, situated in the back of the sacrum stabilizes the joints of the spine. The specific muscles were chosen according to Surface ElectroMyoGraphy for the Non-Invasive Assessment of Muscles (SENIAM) recommendations for sensor locations on neck and trunk muscles (Hermens et al., 1999).

Prior to electrode placement, the skin was prepared by cleansing and abrading with a disinfectant (74.1% ethanol, 10% propanol). If necessary, hair was shaved at specific sites. A double-sided adhesive skin interface was applied on each sensor to firmly stick it to the selected site. A surface EMG sample was collected at a sampling rate of 1.1 kHz using 12 Ag TrignoTM IMU sensors. A bandpass filter of 20–450 Hz was applied. The EMG data were recorded using the EMGworks® software (Delsys Europe Ltd, Greater Manchester, United Kingdom).

The EMG recording was started by taking the maximal voluntary contraction (MVC) of each muscle site. The participants were instructed to elicit maximal contraction for 5 s. The MVC was repeated twice for each muscle with a resting period of 1 min in between.

#### Affect Grid

The subjective mood state of the participants was surveyed in an affect grid by crossing one square grid, which was designed to assess the personal affect in a 9 × 9 square grid with dimensions negative to positive on the x-axis and sleepiness to arousal on the y-axis (Russell et al., 1989).

### Procedure

Prior to the experiment, the participants gave written informed consent to participate in the study and filled out questionnaires. As preparation for the experiment part of the study, the electrodes were placed on the specific sites, skin conductance sensors were attached to the fingers, and MVC data were recorded.

For the main part of the experiment, the participants were seated 100 cm across a television (42 inch). They were asked to sit upright during each condition and baseline recording but allowed to lean back between measurements to avoid fatigue. We choose the sitting position because quiet stance is always affected by keeping postural control, which, depending on the task, has been found to increase muscle activity in the back (Donath et al., 2016). We did not expect the time of leaning against the back of the chair to influence muscle activity in the subsequent trial because the participants sat relaxed and did not engage in other muscle-tensioning activities. Each trial started by recording a baseline (baseline 1), while no pictures were presented for 2 min. It was followed by the positive condition (3 min), a 2 min baseline recording (baseline 2), and the negative condition (3 min). The emotion conditions were presented in a counterbalanced order. Finally, a third baseline (baseline 3) was recorded (2 min). After each condition and baseline, a break of 1 min was given. The third condition of a cognitive stressor was collected, which will not be presented in this study, since it was not the focus of the experiment. Before the first trial (pre-baseline), after each condition, and at the end of the experiment (post-baseline), the participants rated their subjective mood state in an affect grid (Russell et al., 1989). For the positive condition, selected positive pictures of the international affective picture system (IAPS) were combined with positive music (“*Eine kleine Nachtmusik*,” W Mozart). For the negative condition, selected negative pictures of the IAPS were combined with negative music (“Adagio in G Minor,” T Albioni) and presented in a slideshow. Both pieces were found to induce mood in healthy participants effectively (Västfjäll, 2001) and were chosen to intensify the emotions triggered by the pictures.

### Analyses

#### Affect Grid

The affect grid was evaluated by assigning ascending numbers from 1 to 9 to each box of the grid, starting with 1 in the lower left corner, which is equivalent to a most negative and sleepy mood state (depression). The mean of the pre- and post-baselines was calculated, and the valence and arousal ratings of each experimental condition were compared to the mean baseline. The Wilcoxon signed-rank test was performed, because the data did not follow a normal distribution (Kolmogorov–Smirnov test: *p* < 0.05).

#### Skin Conductance

In order to eliminate individual differences in responsivity, the resulting amplitudes were normalized in each participant according to the range correction procedure of Lykken et al. (1966). For statistical analysis, a *t*-test was conducted to examine whether the conditions of showing positive and negative pictures affected the level of arousal. The mean values of baselines 1 and 2 and of baselines 2 and 3 were compared with those of the associated experimental condition.

#### Electromyography

Because of low activity, a time window of 5 s with no overlap was chosen to process the raw EMG data. The data were normalized to the corresponding MVC data and root mean square was calculated using MATLAB. All EMG data are presented in % of the MVC. Pre-processed data were manually inspected; the artifacts and the first and last 3 s of every condition or baseline were removed. For this data set, no normal distribution was ensured (Kolmogorov–Smirnov test: *p* < 0.05). Thus, to analyze statistically, the influence of the conditions on muscle activity compared to the resting period a Wilcoxon signed-rank test was performed comparing the mean of baseline 1 and 2 and the mean of baselines 2 and 3 to the associated experimental condition, respectively. To study the intensity of muscle activity in each condition on specific back regions, sensors were clustered by calculating the means according to their placement in the upper (shoulder region: M. trapezius pars descendes and M. trapezius pars transversalis), middle (thorax region: M. trapezius pars ascendens), and lower back (lumbar region: M. longissimus, M. iliocostalis, and M. multifidus). As an ANOVA is relatively robust against violation of the normal distribution of the population (Schmider et al., 2010; Blanca et al., 2017), an ANOVA with repeated measurements was performed using the region (shoulder, thorax, and lumbar back) as a fixed factor and the condition as a dependent variable.

Additionally, the delta values of muscle activity (EMG) (i.e., experimental condition minus the average of the two baselines) were correlated with the delta values of the valence and arousal state ratings (affect grid).

## Results

Preliminarily, all data were analyzed for normal distribution by performing the Kolmogorov–Smirnov test. All results were Greenhouse–Geisser corrected. *Post-hoc* analyses results were estimated and multiple *p*-values were alpha-corrected according to the Bonferroni correction analysis.

### Affect Grid

Alpha error accumulation was corrected according to Bonferroni correction analysis and the adjusted alpha levels were set to *p* = 0.013, ($p=\frac{\alpha }{n}$). A conducted Wilcoxon signed-rank test comparing the subjective mood ratings after emotion induction to the baseline ratings revealed a significant difference of valence between the negative emotion condition and its baseline: *z* = −4.66, *p* < 0.01. A *post-hoc* analysis with G^*^ power (Faul et al., 2007) revealed a power value (1–ß) of 1 and an effect size of Cohen's *d* = 1.88. The participants rated their mood state after the negative emotion condition significantly more negative (mean ± SD: 4.03 ± 1.58), 95% CI [3.45, 4.61] than after the baseline (6.42 ± 0.85), 95% CI [6.11, 6.73]. No significant difference was found between the valence ratings of the positive emotion condition (6.94 ± 1.21), 95% CI [6.49, 7.38] and its baseline (6.42 ± 0.85), 95% CI [6.11, 6.73]: *z* = −1.93, *p* = 0.053, G^*^ power (1–ß) (Faul et al., 2007) of 0.6 and Cohen's *d* = 0.5. No significant difference was found for arousal ratings after both conditions, positive emotion (5.35 ± 1.89), 95% CI [4.66, 6.05] and its baseline (5.35 ± 1.76), 95% CI [4.71, 6]: *z* = −0.12, *p* = 0.903,G^*^ power (1–ß) (Faul et al., 2007) of 0.1 and Cohen's *d* < 0.001; and negative emotion (4.58 ± 1.63), 95% CI [3.98, 5.98] and its baseline (5.35 ± 1.76), 95% CI [4.71, 6]: *z* = −2.28, *p* = 0.023, G^*^ power (1–ß) (Faul et al., 2007) of 0.5 and Cohen's *d* = 0.45 (see Table 1).

**Table 1:** Means (SD) of subjective mood ratings (affect grid) after experimental conditions (positive and negative) and baseline.

**Positive emotion condition**				**Negative emotion condition**				**Baseline**			
**Valence**		**Arousal**		**Valence**		**Arousal**		**Valence**		**Arousal**	
**M**	**SD**	**M**	**SD**	**M**	**SD**	**M**	**SD**	**M**	**SD**	**M**	**SD**
6.94	1.21	5.35	1.89	4.03	1.58	4.58	1.63	6.42	0.85	5.35	1.76

*Baseline, mean of pre- and post-baselines*.

### Skin Conductance

**Figure 1:**
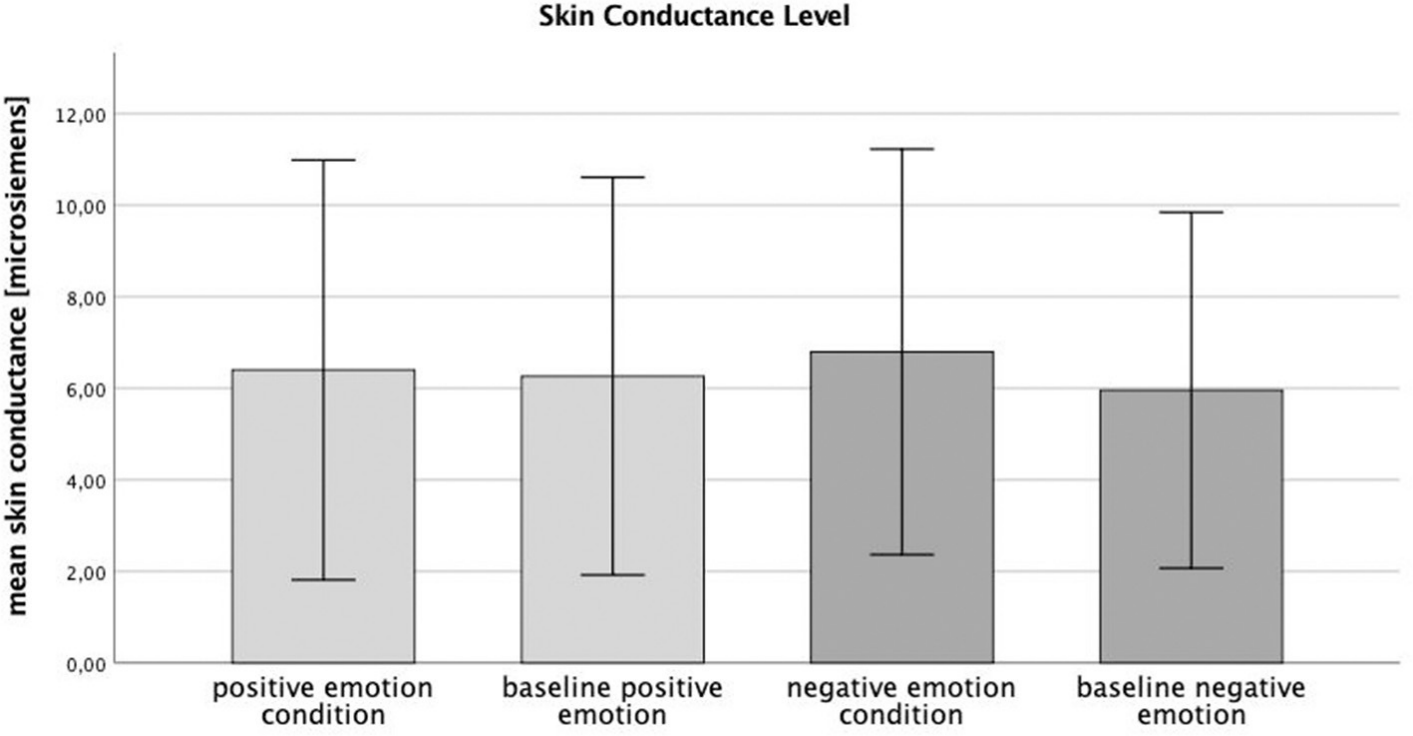
Skin conductance level (microsiemens) during experimental conditions (positive and negative) and baselines.

Alpha error accumulation was corrected according to the Bonferroni correction analysis and the adjusted alpha levels were set to *p* = 0.025, ($p=\frac{\alpha }{n}$). The *t*-test conducted by comparing each condition with the mean corresponding baselines revealed a significant difference for the negative emotion condition: *t*(30) = −2.88, *p* = 0.007, a *post-hoc* analysis with G^*^ power (Faul et al., 2007) indicated a power value (1–ß) of 0.8 and an effect size of Cohen's *d* = −0.40. The skin conductance increased after negative emotion induction (mean ± SD: 6.79 ± 2.22), 95% CI [5.98, 7.61] compared with baseline (5.96 ± 1.94), 95% CI [5.24, 6.67]. No significant difference was found between the skin conductance level after the induction of the positive emotion condition (6.4 ± 2.29), 95% CI [5.56, 7.24] and its baseline (6.26 ± 2.17), 95% CI [5.47, 7.06]: *t*(30) = −1.10, *p* = 0.279, G^*^ power (1–ß) (Faul et al., 2007) of 0.1 and Cohen's *d* = −0.06 (see Figure 1).

### Electromyography

**Figure 2:**
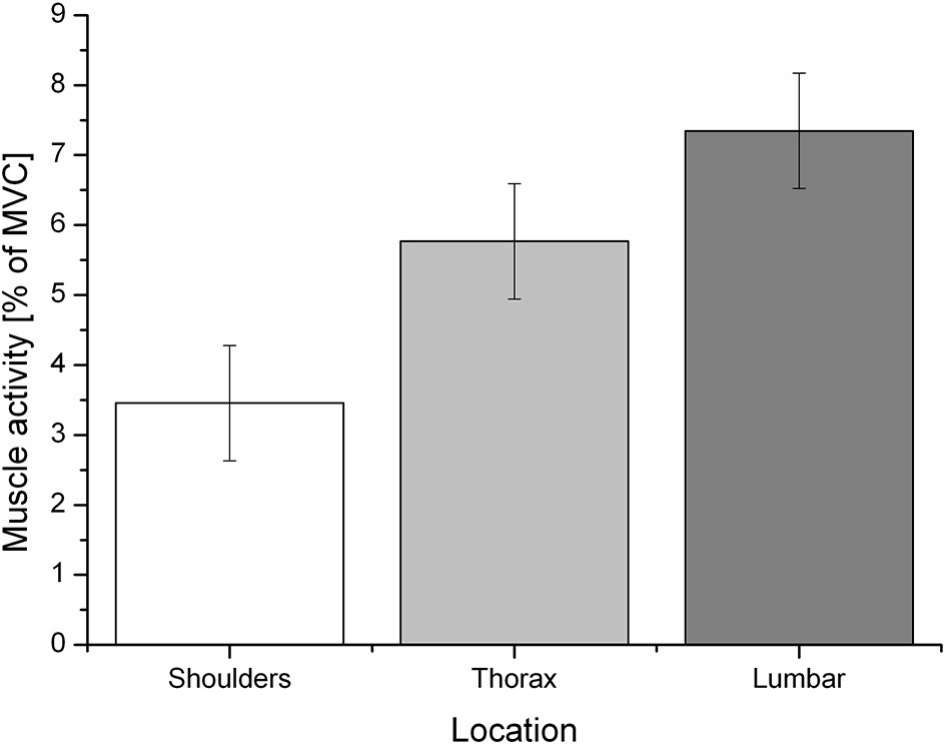
Mean muscle activity (% of MVC) during the negative emotion condition for the specific regions: shoulders, thorax, and lumbar region.

The alpha error accumulation was corrected according to the Bonferroni correction analysis and the adjusted alpha levels were set to *p* = 0.025, ($p=\frac{\alpha }{n}$). The applied Wilcoxon signed-rank test, conducted by comparing the percentage of the MVC of each muscle, revealed a significant difference between the negative emotion condition (mean ± SD: 5.8 ± 11.67), 95% CI [4.59, 7.01] and its corresponding baseline (5.35 ± 4.2), 95% CI [4.91, 5.79]: *z* = −3.51, *p* < 0.01, a *post-hoc* analysis with G^*^ power (Faul et al., 2007) revealed a power value (1–ß) of 0.6 and an effect size of Cohen's *d* = −0.05. No difference was found between the positive emotion condition (5.37 ± 6.03), 95% CI [4.74, 5.99] and its baseline (5.33 ± 4.28), 95% CI [4.89, 5.77]: *z* = −1.50, *p* = 0.134, G^*^ power (1–ß) (Faul et al., 2007) of 0.1 and Cohen's *d* = −0.01. Since the positive emotion condition did not differ from its baseline, it will not be considered for further analysis. The negative emotion condition had a significant effect on the different muscular regions (shoulder, thorax, and lumbar back) *F*_(2, 90)_ = 5.63, *p* = 0.005, partial η^2^ = 0.115 (see Figure 2). The Bonferroni correction analysis revealed a significant difference between the shoulder region (mean ± SD: 3.37 ± 3.34), 95% CI [1.82, 5.09] and the lumbar region (7.19 ± 5.97), 95% CI [5.71, 8.98]: mean difference = −3.89, *p* = 0.004.

The Spearman correlation indicated no significant correlations between muscle activity and mood ratings (affect grid). A significant positive correlation was found between the arousal ratings after the induction of the positive emotion condition and the arousal ratings after induction of the negative emotion condition, *r*_s_(28) = 0.61, *p* < 0.01 (see Table 2).

**Table 2:** Spearman correlations between delta values of muscle activity (% of MVC) and mood ratings (affect grid).

**Variable**	***n***	**M**	**SD**	**1**	**2**	**3**	**4**	**5**	**6**
1. Muscle activity positive emotion	31	0.26	1.01	–					
2. Muscle activity negative emotion	31	−0.30	3.25	−0.16	–				
3. Valence ratings positive emotion	31	0.52	1.29	0.22	0.04	–			
4. Valence ratings negative emotion	31	−2.39	1.38	−0.00	−0.32	−0.30	–		
5. Arousal ratings positive emotion	31	0.00	2.37	−0.06	−0.04	0.05	−0.03	–	
6. Arousal ratings negative emotion	31	−0.77	1.78	−0.00	−0.26	0.04	0.01	0.61^**^	–

** *p < 0.01*.

## Discussion

The aim of this study was to investigate the influence of emotions on different muscular regions in the back. Muscle pattern activities were measured during the presentation of a composition of emotional pictures and music. The EMG data showed significant increased muscular activity during exposure to negative visual and acoustic stimuli. However, no significant differences in muscle activity were found for the positive emotion condition.

These results partly support the hypothesis that, according to the sensorimotor simulation hypothesis, perceived emotions lead to a corresponding motor response. Above that, the results corroborate those of previous research studies, which found emotions to affect subtle physiological reactions, as approach and avoidance behavior. For instance, a sit-to-walk movement has been found to decelerate during the consolidation of a sad memory, whereas it accelerate when an incidence of joy or anger was remembered (Kang and Gross, 2015). The fact that only negative emotions led to physiological change goes in line with a study that investigated body movements during walking. Depressed or with sadness induced participants, walk with decreased speed, arm swing, and vertical head movements, compared to healthy or happy participants (Michalak et al., 2009), whereas the sitting posture has been affected by positive and negative emotions. Positive feelings cause a more upright sitting position, whereas emotionally negative feelings lead to less upright sitting (Oosterwijk et al., 2009). In contrast, the positive emotion condition in this study seemed affected the emotional state of the participants at all, a fact which was shown by the methodical control: skin conductance significantly increased only during induction of the negative emotion condition. Previous research has shown that skin conductance responds to high-arousing external stimuli and indicates a change in physiological state (Horslen and Carpenter, 2011). The findings on muscular activity and skin conductance are further supported by subjective mood ratings, which found only a significant difference in the valence ratings between the negative emotion condition and its baseline. No difference was found in the arousal ratings. Positive emotion induction did not change any of the mood ratings, which indicates the ineffectiveness of the positive stimuli. Apparently, all the conditions were perceived as equally arousing because the delta values correlated positively, which stands in contrast to the assumption of Russell et al. (1989).

As the slumped sitting position has been found to increase muscle activities differently (Caneiro et al., 2010), we investigated emotional impressions in three different muscular regions: shoulder, thorax, and lumbar. Again, the hypothesis could only partly be confirmed. During the induction of the negative emotion condition, muscle activity significantly increased in the lumbar region compared with the shoulder region. An explanation could be a less upright sitting position, as negative emotions were found to decrease posture height (Oosterwijk et al., 2009), and movements of the lumbar region increase specific muscle activity (Castanharo et al., 2014). This interpretation, however, needs further investigations. The fact that we did not find any increased muscle activity in the shoulder region might be because of the experiment setup, which asked the participants to sit motionless and upright. While slumping of the thorax and shoulder regions might be more noticeable, small movements of the lumbar region could have happened unconsciously. In contrast to Oosterwijk et al. (2009), who used *disappointment* as a negative emotion, we suggest that the composition of negative visual stimuli and music did not exclusively induce one emotion quality and could have elicited a broader spectrum of negative emotions. For future research, it would be essential to study the interrelation between muscular activity in the back, elicited through emotions, and sitting posture, and, further, the extent to which a more upright or slumped sitting posture could be recognized by others.

The fact that muscle activity did not correlate with subjective mood ratings seems surprising. It has, however, been reported by several studies, most of which investigated perceived stress (Ekberg et al., 1995) or pain levels (Sjörs et al., 2009; Strøm et al., 2009). It could be argued that individuals perceive and express emotions differently, depending on their personal traits (Servián-Franco et al., 2015).

Put together, the results of this study show that negative emotions, elicited through negative visual and acoustic stimuli, lead to increased differential muscle activity in the back. We suggest that this subtle physiological change is the first indication of emotional expressions through the sitting posture, whether it is consciously recognizable or not. Similarly, previous research has found other consciously non-recognizable emotional expressions to be mimicked by interacting partners, i.e., pupil dilation (Kret et al., 2014). The findings of this study support the concept of sensorimotor simulation process – perceiving an emotion leads to own emotional expression of the same emotion, which, if the concept holds, could also activate an unrelated emotional expression compared with the observed one. Above that, it corroborates the notion of a reciprocal relationship between emotional state and bodily reactions, as proposed by the embodiment theories (Michalak et al., 2009), which, if expressed through any physiological reaction can be seen as being part of the greater question of social communication.

## Limitations

The study is limited by the experiment setup, which investigated muscular activity but did not control for visible postural changes in the sitting position. Thus, it could not be clarified whether the muscular activity was related to a more upright or more slumped sitting posture. Interesting for future research could also be to test a similar setup but in the standing position, which would allow to investigate a connection between muscle activity in the back and approach and avoidance behavior in response to emotional stimuli. Besides, the study is limited by the small sample size of participants, and further by the positive emotion condition, which failed to affect any methodological measurements, i.e., the muscle activity, level of skin conductance, or subjective mood ratings of the participants did not significantly change after exposure to the positive emotion condition. Thus, the positive emotion condition should be revised with other stimuli.

## Data Availability Statement

The datasets generated for this study are available on request to the corresponding author.

## Ethics Statement

This study was performed in line with the principles of the Declaration of Helsinki. Approval was granted by the Ethics Committee of the University of Regensburg (Date 12-11-2019/No. 19-1625-101). The participants provided their written informed consent to participate in this study.

## Author Contributions

CS was responsible for data acquisition, analyses of the data, and text writing of the manuscript. CS, SK, and PJ planned and discussed the experiment setup. PJ supported and supervised CS from the whole process of creating an experiment to concluding the results and writing the manuscript. SD provided the measuring instruments and laboratory. All authors contributed to the article and approved the submitted version.

## Conflict of Interest

The authors declare that the research was conducted in the absence of any commercial or financial relationships that could be construed as a potential conflict of interest.
